# Supplementary material for: Wastewater sequencing reveals community and variant dynamics of the collective human virome
Source: Nat Commun. 2023 Oct 28;14:6878. doi: 10.1038/s41467-023-42064-1 (PMC10613200; doi:10.1038/s41467-023-42064-1)
Supplement: Supplementary file 1 — Supplementary Information [file 41467_2023_42064_MOESM1_ESM.pdf]

**Fig. S1**

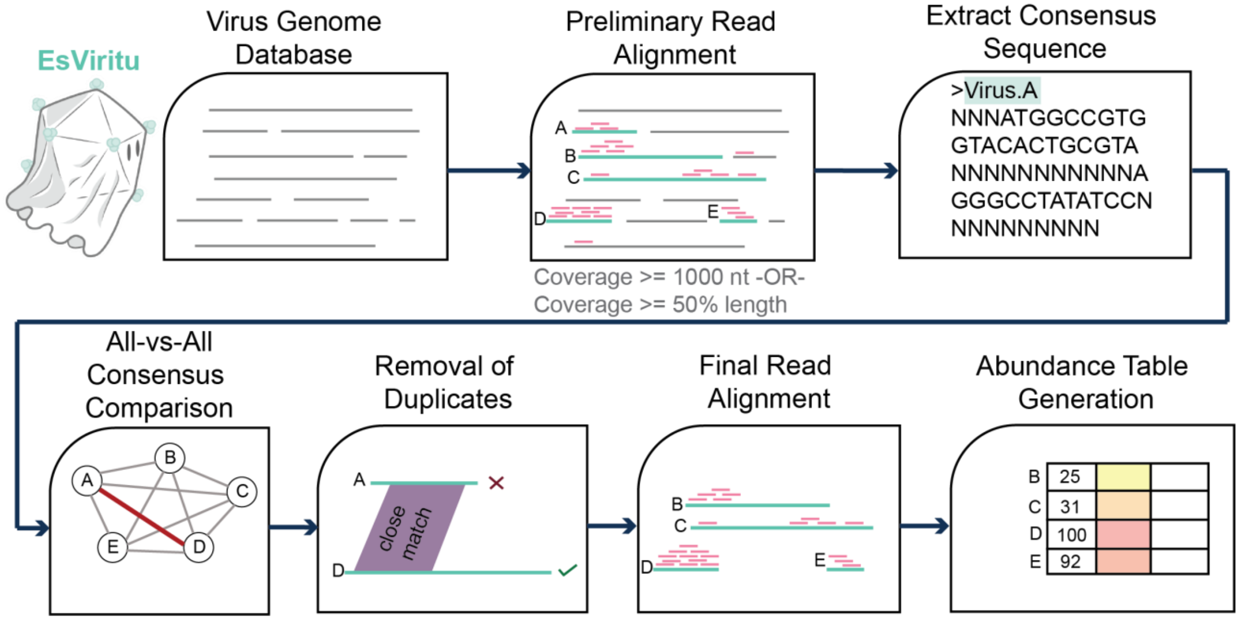


**Schematic of EsViritu pipeline.** EsViritu is suitable for detecting known and near-known mammalian virus genome sequences in metagenomic data. Relying on a de-replicated database of virus genomes, EsViritu aligns input reads to virus genomes with minimap2/coverM. For genomes that have read coverage of at least 1000 nucleotides or 50% of the sequence length, consensus sequences are extracted with SAMtools. Consensus sequences undergo all-vs-all pairwise comparison with BLASTn. Highly similar consensus sequences are de-replicated into the best (longest) exemplar. Reads are re-aligned to the de-replicated reference sequences with minimap2/coverM to calculate virus abundance metrics.

**Fig. S2**

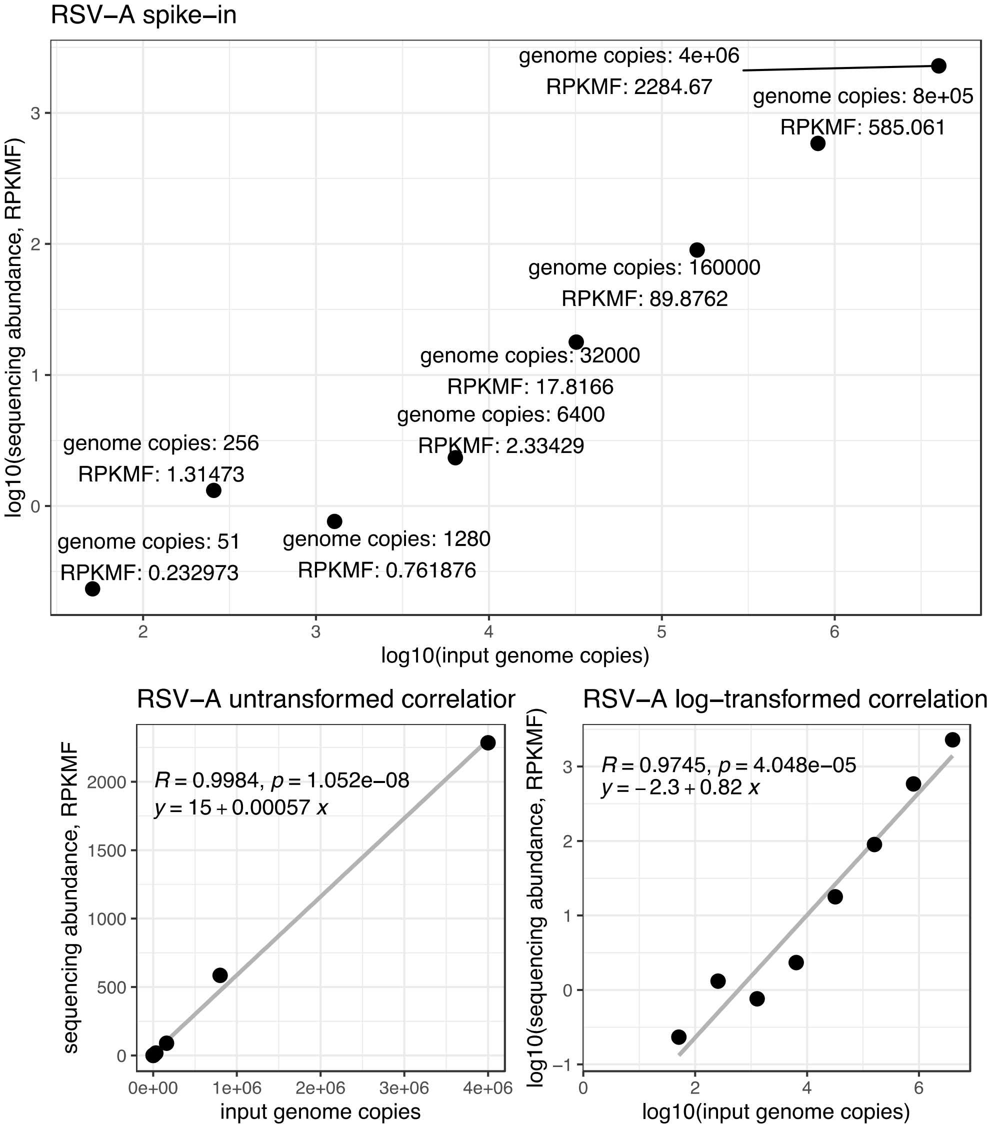


**Dynamic range of pathogen detection in wastewater using RSV spike-in.** All charts represent the results of an experiment spiking RSV into real wastewater samples. Wastewater samples were first processed with the hybrid-capture method described in this paper to screen for presence of RSV and only RSV-negative samples were used here. A 5:1 dilution series, starting with 4 million estimated genome copies down to 51 estimated genome copies, was prepared with wastewater samples, then processed with hybrid capture methods, sequenced, and quantified with EsViritu.

## Fig. S3

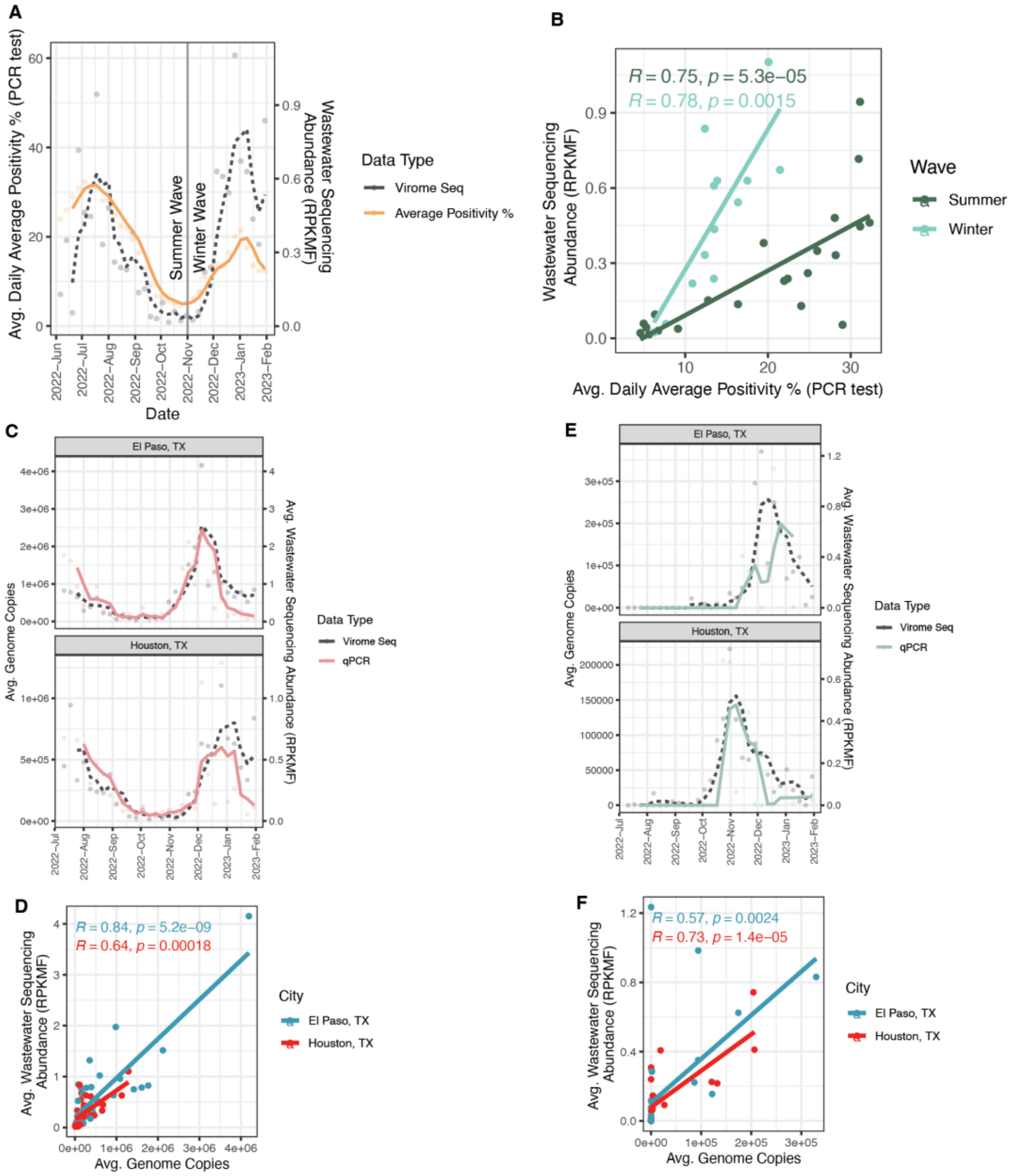


**Comparison of virome sequencing to clinical data and qPCR of wastewater samples. (A)** SARS-CoV-2 wastewater sequencing abundance compared to percent positivity of PCR tests in Houston, TX. **(B)** Scatter plot and correlation between wastewater sequencing abundance compared to percent positivity of PCR tests of SARS-CoV-2 in Houston, TX. **(C)** SARS-CoV-2 wastewater sequencing abundance compared qPCR values in Houston and El Paso, TX. **(D)** Scatter plot and correlation between wastewater sequencing abundance compared to qPCR values of SARS-CoV-2 in Houston and El Paso, TX. **(E)** Influenza A wastewater sequencing abundance compared qPCR values in Houston and El Paso, TX. **(F)** Scatter plot and correlation between wastewater sequencing abundance compared to qPCR values of Influenza A in Houston and El Paso, TX.

**Fig. S4**

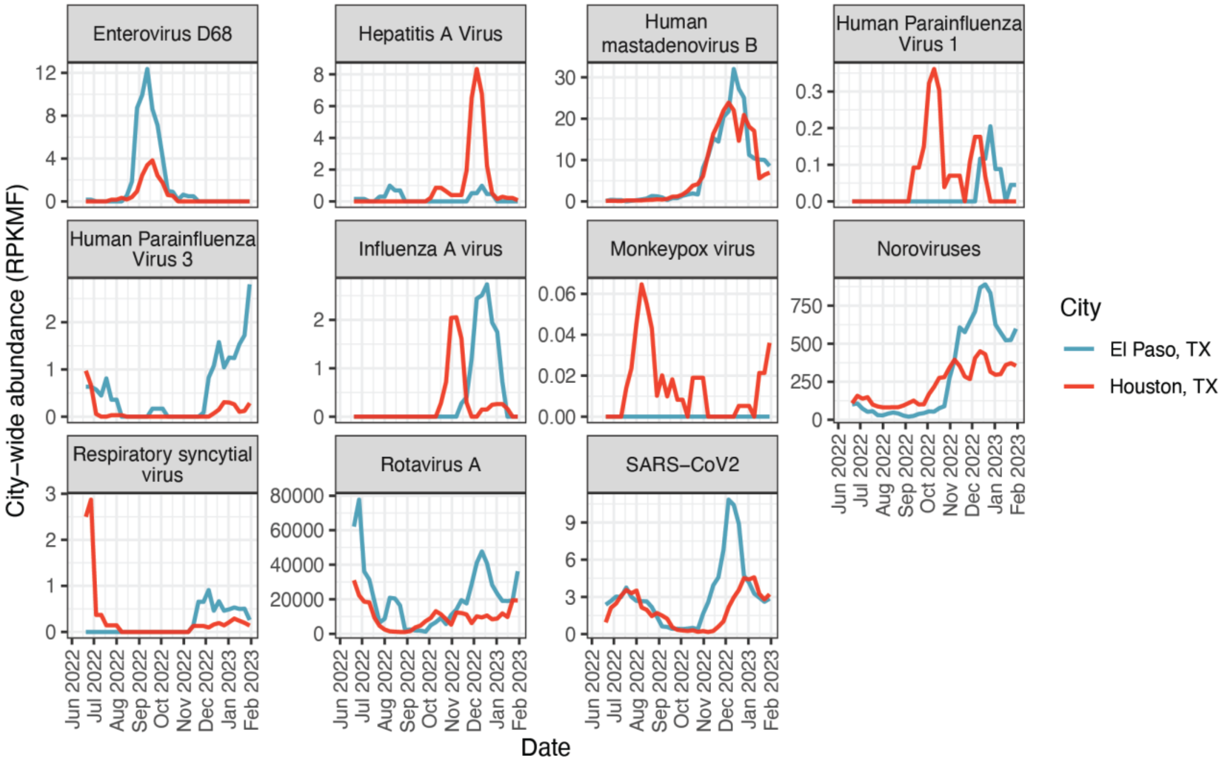


**Virome sequencing Abundance of Major Pathogens.** Moving average charts of eight virus pathogens in Houston and El Paso, TX.

**Fig. S5**

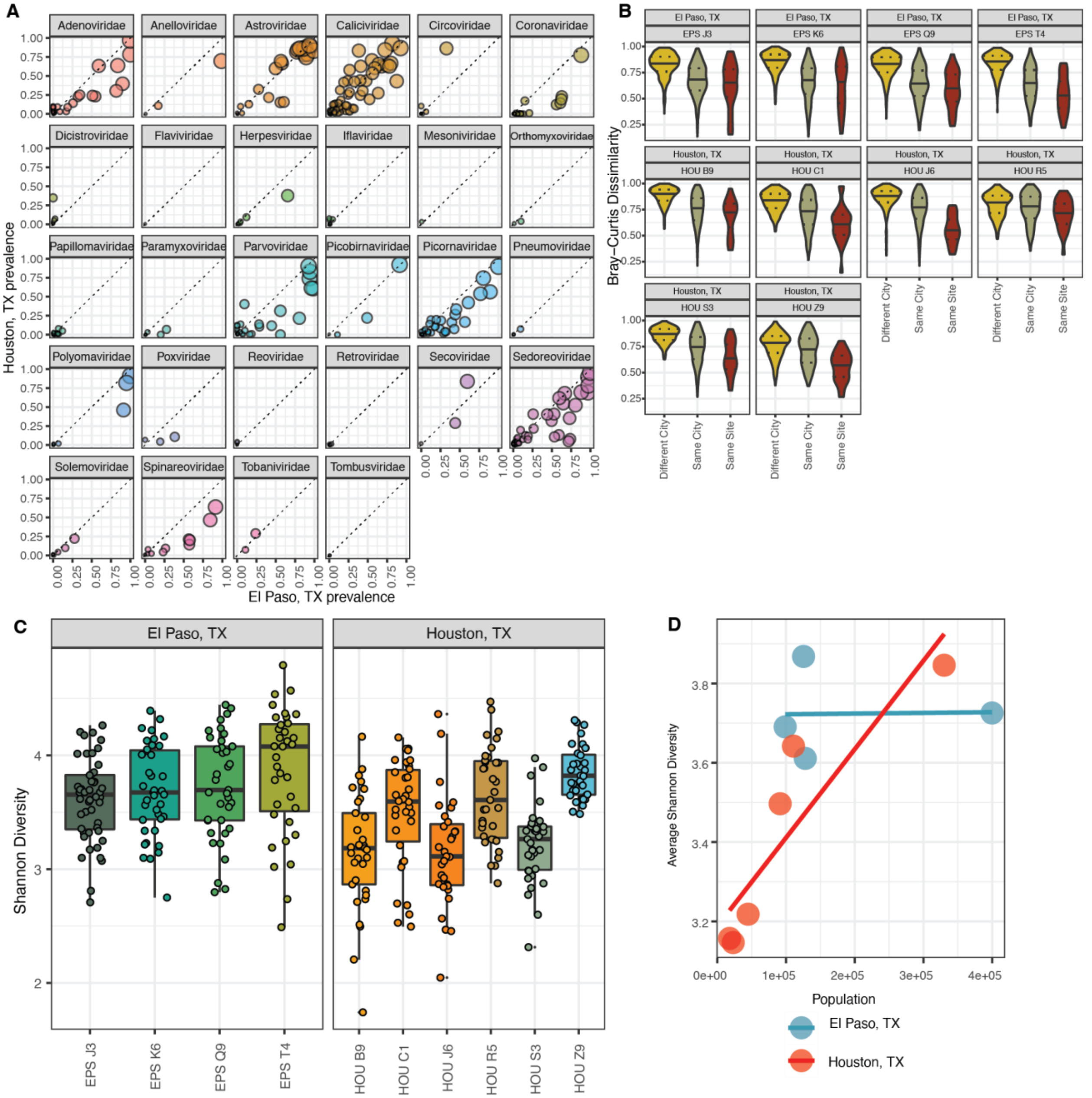


**Additional wastewater virome community metrics. (A)** All detected virus strains faceted by family with prevalence in Houston and El Paso. **(B)** Bray-Curtis dissimilarity between samples taken +/- seven days apart, comparing samples from the same site, different site but same city, and different city, faceted by site. **(C)** Shannon diversity by site. Boxplots are defined as: center line = median, lower and upper box-bounds = 25$^{th}$ and 75$^{th}$ data percentiles, and whiskers extend to the minimum and maximum values within 1.5 interquartile ranges. **(D)** Comparison of average Shannon diversity of wastewater treatment plants and population of catchment areas.

**Fig. S6**

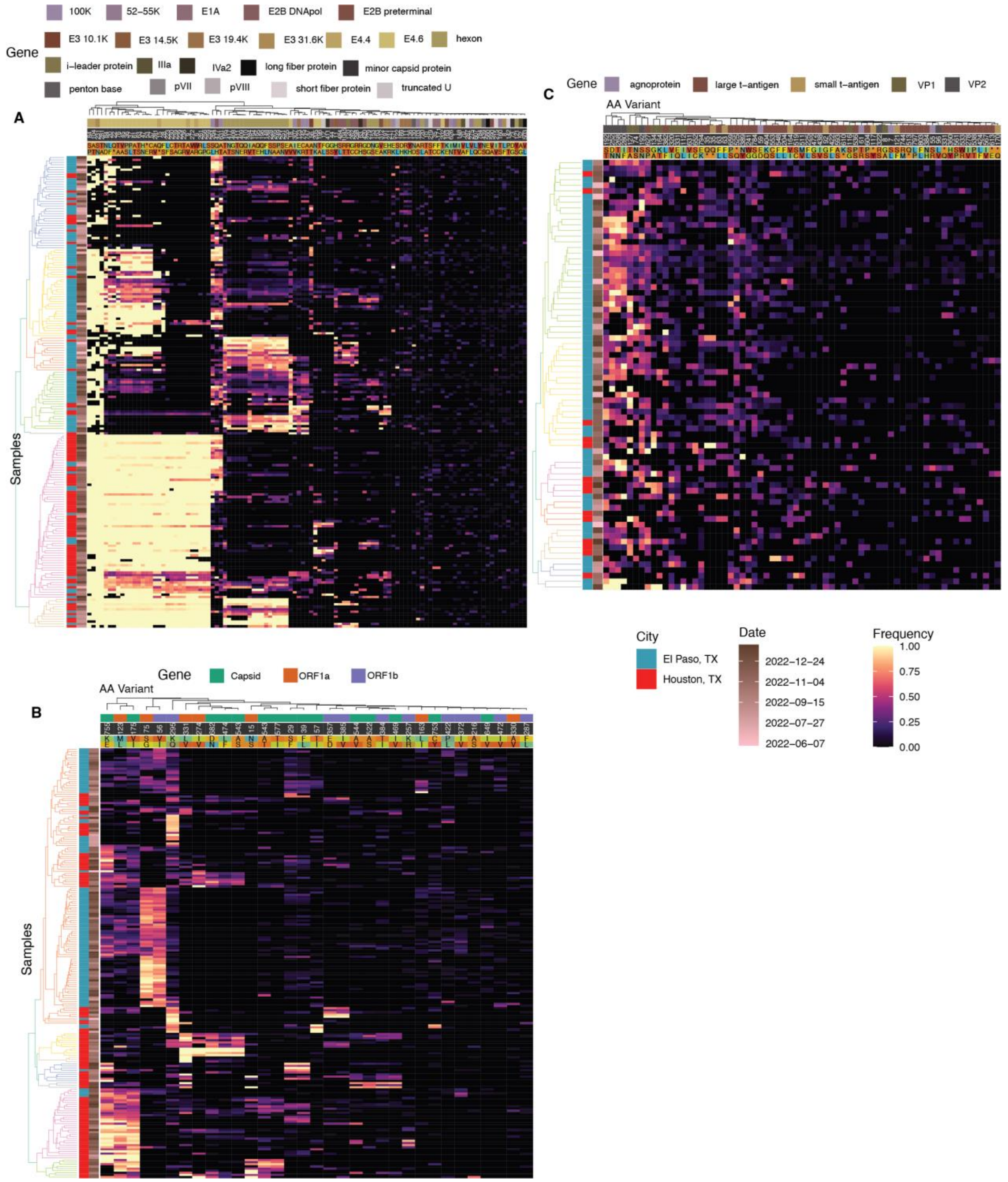


**Evaluation of non-synonymous variants in prevalent wastewater viruses (continued). (A)** Dendrogram and heatmap describing frequency of common non-synonymous variants of astrovirus MLB1 by sample. **(B)** Like (A) but with Human Adenovirus 41. **(C)** Like (A) but with JC Polyomavirus.

**Supplemental Table 1:** Sequences and concentration of Influenza A, SARS-CoV-2 and RSV-A primers and probes standards used in RT-qPCR assay.

| Primer | Sequence (5’-3’) | Concentration |
|---|---|---|
| InfA Forward1 | 5’-CAAGACCAATCYTGTCACCTCTGAC-3’ | 3.3 µM |
| InfA Forward2 | 5’-CAAGACCAATYCTGTCACCTYTGAC-3’ | 3.3 µM |
| InfA Reverse1 | 5’-GCATTYTGGACAAAVCGTCTACG-3’ | 5 µM |
| InfA Reverse2 | 5’-GCATTTTGGATAAAGCGTCTACG-3’ | 1.67 µM |
| InfA probe | 5’-/FAM/TGCAGTCCT/ZEN/CGCTCACTGGGCACG/3IABkFQ/-3’ | 1.67 µM |
| SC2-Forward | 5’-CTGCAGATTTGGATGATTTCTCC-3’ | 6.67 µM |
| SC2-Reverse | 5’-CCTTGTGTGGTCTGCATGAGTTTAG-3’ | 6.67 µM |
| SC2-Probe | 5’-/TexRd-XN/ATTGCAACA/TAO/ATCCATGAGCAGTGCTGA CTC/3IAbRQSp/-3’ | 1.67 µM |
| RSV-A forward | 5’-GATACACTCAACAAAGATCAACTTCTGTCA-3’ | 0.4 µM |
| RSV-A reverse | 5’-AGGAGTGTCAATGCTGTCTCCTGTG-3’ | 0.4 µM |
| RSV-A probe | 5’-/FAM/TCCAGCAAA/ZEN/TACACCATCCAACGGAG/3IABkFQ/-3’ | 0.2 µM |
| Influenza A oligonucleotide standard | 5’-aatggctaaagacaagaccaatcctgtcacctctgactaa ggggattttaggatttgtgttcacgctcaccgtgcccagt gagcgaggactgcagcgtagacgctttgtccaaaatgccctcaatgggaa-3’ | |
| RSVA oligonucleotide standard | 5’-GAATGATACACTCAACAAAGATCAACTTCTGTCATCCAGCAAAT ACACCATCCAACGGAGCACAGGAGACAGCATTGACACTCCT-3’ | |
