# Supplementary material for: Wastewater sequencing reveals community and variant dynamics of the collective human virome
Source: Nat Commun. 2023 Oct 28;14:6878. doi: 10.1038/s41467-023-42064-1 (PMC10613200; doi:10.1038/s41467-023-42064-1)
Supplement: Supplementary file 3 — Reporting Summary [file 41467_2023_42064_MOESM3_ESM.pdf]

nature portfolio

Corresponding author(s): Anthony W. Maresso, Eric Boerwinkle, Joseph F. Petrosino

Last updated by author(s): Sep 19, 2023

Reporting Summary

Nature Portfolio wishes to improve the reproducibility of the work that we publish. This form provides structure for consistency and transparency in reporting. For further information on Nature Portfolio policies, see our Editorial Policies and the Editorial Policy Checklist.

Statistics

For all statistical analyses, confirm that the following items are present in the figure legend, table legend, main text, or Methods section.

n/a	Confirmed	
☐	☒	The exact sample size (*n*) for each experimental group/condition, given as a discrete number and unit of measurement
☐	☒	A statement on whether measurements were taken from distinct samples or whether the same sample was measured repeatedly
☐	☒	The statistical test(s) used AND whether they are one- or two-sided<br>*Only common tests should be described solely by name; describe more complex techniques in the Methods section.*
☒	☐	A description of all covariates tested
☐	☒	A description of any assumptions or corrections, such as tests of normality and adjustment for multiple comparisons
☐	☒	A full description of the statistical parameters including central tendency (e.g. means) or other basic estimates (e.g. regression coefficient) AND variation (e.g. standard deviation) or associated estimates of uncertainty (e.g. confidence intervals)
☐	☒	For null hypothesis testing, the test statistic (e.g. *F*, *t*, *r*) with confidence intervals, effect sizes, degrees of freedom and *P* value noted<br>*Give P values as exact values whenever suitable.*
☒	☐	For Bayesian analysis, information on the choice of priors and Markov chain Monte Carlo settings
☒	☐	For hierarchical and complex designs, identification of the appropriate level for tests and full reporting of outcomes
☐	☒	Estimates of effect sizes (e.g. Cohen's *d*, Pearson's *r*), indicating how they were calculated

Our web collection on statistics for biologists contains articles on many of the points above.

Software and code

Policy information about availability of computer code

Data collection	No software was used for data collection
Data analysis	EsViritu v0.1.1<br>https://github.com/cmmr/EsViritu<br>custom code: https://github.com/cmmr/TX_wastewater_virome<br>NCBI datasets<br>CheckV<br>Taxonkit<br>BBtools<br>Vegan<br>ggplot<br>ggpubr<br>RtSNE<br>iVar<br>ggtree<br>aplot<br>tidyquant

For manuscripts utilizing custom algorithms or software that are central to the research but not yet described in published literature, software must be made available to editors and reviewers. We strongly encourage code deposition in a community repository (e.g. GitHub). See the Nature Portfolio guidelines for submitting code & software for further information.

Data

Policy information about availability of data

All manuscripts must include a data availability statement. This statement should provide the following information, where applicable:

- Accession codes, unique identifiers, or web links for publicly available datasets
- A description of any restrictions on data availability
- For clinical datasets or third party data, please ensure that the statement adheres to our policy

The data used in this study's analyses is deposited at Zenodo repository https://zenodo.org/record/7884454, doi: 10.5281/zenodo.7884454 (follow instructions on the GitHub repository). All sequencing reads are uploaded to SRA at accession PRJNA966185 (https://www.ncbi.nlm.nih.gov/bioproject/?term=PRJNA966185) with any human sequences removed.

Research involving human participants, their data, or biological material

Policy information about studies with human participants or human data. See also policy information about sex, gender (identity/presentation), and sexual orientation and race, ethnicity and racism.

Reporting on sex and gender	*Use the terms sex (biological attribute) and gender (shaped by social and cultural circumstances) carefully in order to avoid confusing both terms. Indicate if findings apply to only one sex or gender; describe whether sex and gender were considered in study design; whether sex and/or gender was determined based on self-reporting or assigned and methods used. Provide in the source data disaggregated sex and gender data, where this information has been collected, and if consent has been obtained for sharing of individual-level data; provide overall numbers in this Reporting Summary. Please state if this information has not been collected. Report sex- and gender-based analyses where performed, justify reasons for lack of sex- and gender-based analysis.*
Reporting on race, ethnicity, or other socially relevant groupings	*Please specify the socially constructed or socially relevant categorization variable(s) used in your manuscript and explain why they were used. Please note that such variables should not be used as proxies for other socially constructed/relevant variables (for example, race or ethnicity should not be used as a proxy for socioeconomic status). Provide clear definitions of the relevant terms used, how they were provided (by the participants/respondents, the researchers, or third parties), and the method(s) used to classify people into the different categories (e.g. self-report, census or administrative data, social media data, etc.) Please provide details about how you controlled for confounding variables in your analyses.*
Population characteristics	*Describe the covariate-relevant population characteristics of the human research participants (e.g. age, genotypic information, past and current diagnosis and treatment categories). If you filled out the behavioural & social sciences study design questions and have nothing to add here, write "See above."*
Recruitment	*Describe how participants were recruited. Outline any potential self-selection bias or other biases that may be present and how these are likely to impact results.*
Ethics oversight	*Identify the organization(s) that approved the study protocol.*

Note that full information on the approval of the study protocol must also be provided in the manuscript.

Field-specific reporting

Please select the one below that is the best fit for your research. If you are not sure, read the appropriate sections before making your selection.

☐ Life sciences ☐ Behavioural & social sciences ☒ Ecological, evolutionary & environmental sciences

For a reference copy of the document with all sections, see nature.com/documents/nr-reporting-summary-flat.pdf

Ecological, evolutionary & environmental sciences study design

All studies must disclose on these points even when the disclosure is negative.

Study description	Influent wastewater was collected at wastewater treatment plants in Houston and El Paso, TX, USA. Samples were processed, nucleic acid was extracted. Oligonucleotide probes were used to enrich human and animal virus DNA/RNA, then the nucleic acid was sequenced using massively parallel technology. Viral sequences were analyzed for clinical correlation and virome community attributes.
Research sample	wastewater sample: Between 100-500 mL of raw wastewater collected over a 24-hour period. These samples were chosen to represent the viruses of a wastewater treatment plant catchment area population during time of collection.
Sampling strategy	Sampling was conducted weekly for ~9 months at 10 separate wastewater treatment plants. Sample size calculations were not performed, and sample size was determined by a) budgetary allotment, and b) cooperation of wastewater treatment plant professionals in Houston and El Paso.

Data collection	Sequencing data was collected by MCR and SCJ and recorded in internal lab information management system. Sequence data are uploaded to SRA.
Timing and spatial scale	Data was collected from May 2022 - February 2023 in El Paso and Houston, TX, USA. This was the largest effort of its kind, as far as the authors are aware. The collection began when the protocol for virome sequencing of wastewater was developed by the authors, and was concluded (for this study) when "winter waves" of SARS-CoV-2 and Influenza Virus were waning. This allowed comparison to clinical data sets.
Data exclusions	No data were excluded
Reproducibility	The data correlated well with clinical reporting of SARS-CoV-2, Influenza virus, and Monkeypox virus. In some ways, the data are not reproducible, as they rely on wastewater samples that are captured at a particular place and time. On the other hand, we track the number of different virus species detected by site over time and compare virome communities to recent samples (see manuscript) to measure similarity.
Randomization	This is not relevant for our study as the aim was to understand the importance of spatio-temporal factors. Unlike most experiments in the clinic or the lab, we could not randomly split wastewater treatment samples into treatment and control groups.
Blinding	This was not relevant as the main metadata that needed to be used (dates/locations) in analyses reveal the identity of the samples

Did the study involve field work? ☐ Yes ☒ No

Reporting for specific materials, systems and methods

We require information from authors about some types of materials, experimental systems and methods used in many studies. Here, indicate whether each material, system or method listed is relevant to your study. If you are not sure if a list item applies to your research, read the appropriate section before selecting a response.

Materials & experimental systems

n/a	Involved in the study
☒	☐ Antibodies
☐	☒ Eukaryotic cell lines
☒	☐ Palaeontology and archaeology
☒	☐ Animals and other organisms
☒	☐ Clinical data
☒	☐ Dual use research of concern
☒	☐ Plants

Methods

n/a	Involved in the study
☒	☐ ChIP-seq
☒	☐ Flow cytometry
☒	☐ MRI-based neuroimaging

Eukaryotic cell lines

Policy information about cell lines and Sex and Gender in Research

Cell line source(s)	Hep-2 Cells: sourced from ATCC, catalog # CCL-23. Homo sapiens, female
Authentication	Hep-2 cells were authenticated by ATCC but were not authenticated in our laboratory
Mycoplasma contamination	Hep-2 cells were not tested for Mycoplasma contamination
Commonly misidentified lines (See ICLAC register)	*Name any commonly misidentified cell lines used in the study and provide a rationale for their use.*
